# DCE-MRI, DW-MRI, and MRS in Cancer: Challenges and Advantages of Implementing Qualitative and Quantitative Multi-parametric Imaging in the Clinic

**DOI:** 10.1097/RMR.0000000000000103

**Published:** 2016-10-06

**Authors:** Jessica M. Winfield, Geoffrey S. Payne, Alex Weller, Nandita M. deSouza

**Affiliations:** ∗ MRI Unit, The Royal Marsden NHS Foundation Trust, Downs Road, Surrey; † Cancer Research UK Cancer Imaging Centre, Division of Radiotherapy and Imaging, The Institute of Cancer Research, London, UK.

**Keywords:** diffusion-weighted MRI, dynamic contrast-enhanced MRI, magnetic resonance spectroscopy, neoplasms

## Abstract

Multi-parametric magnetic resonance imaging (mpMRI) offers a unique insight into tumor biology by combining functional MRI techniques that inform on cellularity (diffusion-weighted MRI), vascular properties (dynamic contrast-enhanced MRI), and metabolites (magnetic resonance spectroscopy) and has scope to provide valuable information for prognostication and response assessment. Challenges in the application of mpMRI in the clinic include the technical considerations in acquiring good quality functional MRI data, development of robust techniques for analysis, and clinical interpretation of the results. This article summarizes the technical challenges in acquisition and analysis of multi-parametric MRI data before reviewing the key applications of multi-parametric MRI in clinical research and practice.

Magnetic resonance imaging (MRI) has markedly increased in demand as a diagnostic modality over the last decade because it has unparalleled potential to generate tissue contrast on the basis of differences in tissue properties. Information on tissue structure and function can be obtained from measurements of intrinsic differences in tissue relaxation times of hydrogen nuclei (protons) following radiofrequency (RF) excitation, as well as evaluation of fat content, water diffusion, vascularity, elasticity, and characterization of MR-active nuclei by their molecular environment (chemical bonds). In addition, injected extrinsic paramagnetic agents such as gadolinium chelates tend to accumulate in tumors, highlighting their location on T_1_-weighted images. Following the uptake of these agents dynamically probes the tissue vascular state by permitting the generation of semi-quantitative and quantitative parameters relating to tissue perfusion and permeability. A number of contrast-generating techniques can therefore be employed in the same examination to characterize a tissue of interest.

Cancer tissues, with few exceptions, are notably more vascular than their surrounding normal tissues. Because of intense cellular proliferative activity, they also tend to be more densely cellular. This makes dynamic contrast-enhanced MRI (DCE-MRI) combined with diffusion-weighted MRI (DW-MRI) a useful combination of techniques to document these respective features. Certain tumors are also recognizable through a metabolite fingerprint, for example, loss of N-acetyl aspartate (NAA) in gliomas or loss of citrate in prostate cancer, together with an elevation in total choline, which is universal to tumors (Fig. 1). When DCE-MRI, DW-MRI, and magnetic resonance spectroscopy (MRS) are implemented together with morphological T_1_-weighted (T_1_-w) and T_2_-weighted (T_2_-w) imaging, the combination is now commonly referred to as multi-parametric MRI (mpMRI). The purpose of this article is to describe the challenges in data acquisition and analysis of qualitative and quantitative data sets, discuss issues in data interpretation, and examine the role of mpMRI as a decision-making tool in the clinical setting.

**FIGURE 1:**
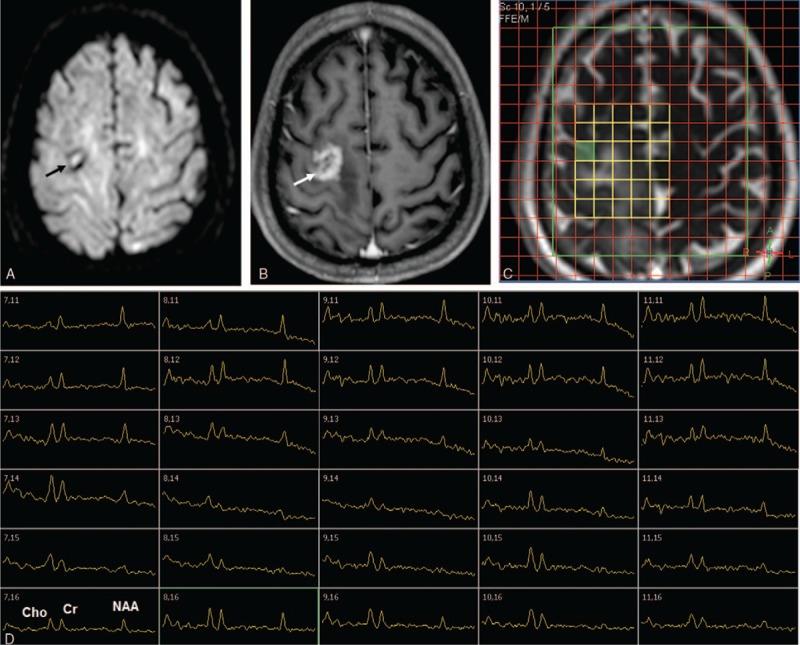
mpMRI examination of a patient with grade IV glioblastoma multiforme of the right parietal lobe (marked by arrows in A and B), acquired on a Philips 3T Achieva TX system. A, Axial diffusion-weighted image (b = 1000 s mm^−2^). B, Axial postcontrast T_1_-w image. C, TruFISP image of same axial slice, with grid showing locations of voxels used in MRSI. D, ^1^H spectra obtained from the voxels shown in (C), showing peaks due to choline (Cho), creatine (Cr), and N-acetyl aspartate (NAA). Data acquired with TR = 1500 ms and TE = 144 ms. Note the relatively high NAA in normal brain, compared with reduced NAA and elevated choline in the tumor region, and that the voxels are metabolically abnormal in regions in which there is no significant uptake of contrast agent (B).

## TECHNICAL CHALLENGES IN DATA ACQUISITION

The main aims in data acquisition are to acquire good quality data [high signal-to-noise ratio (SNR), high spatial resolution, no signal artifacts] in an acceptable total scan time. SNR increases at least in proportion to magnetic field strength.^1^ SNR is also improved with close-fitting external phased array receiver coils, supplemented with internal receiver coils where appropriate, such as for prostate^2,3^ and cervix.^4^ Recently, the move of the analog-to-digital convertors from the equipment room to the magnet housing and even to within the RF coil has greatly improved SNR by reducing noise in the receiver pathway (Philips Medical Systems quote a 40% improvement for their coils, http://www.philips.ng/healthcare/product/HC781342/ingenia-30t-mr-system). Optimum strategies for addition of data from the phased array elements may differ between MRI applications^5^ and MRS.^6^ Shimming of the static magnetic field gives better resolved peaks in MRS scans as well as reducing the susceptibility distortions characteristic of echo-planar imaging (EPI) acquisitions. Such artifacts are further reduced using parallel acquisition strategies.^7^ At higher magnetic fields, multi-transmit systems permit shimming of the B_1_ field that greatly improves uniformity of detection, and can reduce specific absorption rate (SAR).^8^

Combining data from different functional MRI techniques is simplest if they have been acquired using the same geometry (slice thickness etc.). However, MRS in particular has a lower SNR than other functional methods and therefore compromises have to be made depending on the specific system and questions being addressed. Consideration also needs to be given to the order of data acquisition: contrast-enhanced images acquired before MRS aid placement of single-voxel or single-slice 2D-magnetic resonance spectroscopic imaging (MRSI, Fig. 1C) but risk the effects of the contrast agent on some metabolite signals.^9,10^ Use of the same scanner and imaging protocol is recommended for follow-up studies, particularly when deriving quantitative results.

DW-MRI data are usually acquired using EPI, which allows rapid acquisition, although other DW-MRI techniques are possible. EPI is particularly vulnerable to artifacts and careful optimization is required.^11^ Furthermore, because low SNR affects image quality and quantitation, a reduced spatial resolution (eg, 2.5 mm-by-2.5 mm pixels with 5 mm slice thickness) and as short an echo time (TE) as possible are used to maximize SNR. Other strategies to increase SNR include reducing the highest b-value acquired, applying parallel imaging, using monopolar diffusion-encoding gradients, and using diffusion-encoding schemes that apply gradients along >1 direction simultaneously (eg, 3-scan trace, gradient overplus).^12^

Geometric distortions caused by inhomogeneities in the static B_0_ field may be more problematic at 3 T than 1.5 T but can be reduced by using advanced shimming methods and increasing the readout bandwidth. Geometric distortion due to time-varying B_0_ field inhomogeneities caused by eddy currents^13^ can be minimized by reducing the maximum b-value acquired; using a sequence with eddy-current compensation, such as a double spin-echo (DSE)^14^; increasing the readout bandwidth; and using parallel imaging.^11,15^ Ghosting, due to phase-correction errors, can be reduced by optimizing the echo spacing through adjusting the receiver bandwidth and TE. Good suppression of the fat signal is required, as the chemical-shift artifact from unsuppressed fat may obscure areas of interest and bright fat may affect image scaling. To do this, inversion-recovery (IR) or spectral methods may be used (the preferred method may be application- and scanner-dependent); a combination of fat suppression techniques may be required at 3 T.^16^

**FIGURE 2:**
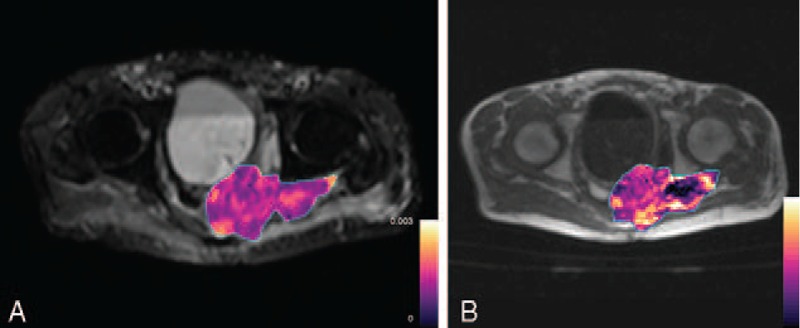
Axial images from a patient with rectal adenocarcinoma. A, ADC map (color) superimposed on b = 0 s/mm^2^ image. B, K^trans^ map (color) superimposed on pre-contrast T_1_-w image.

**FIGURE 3:**
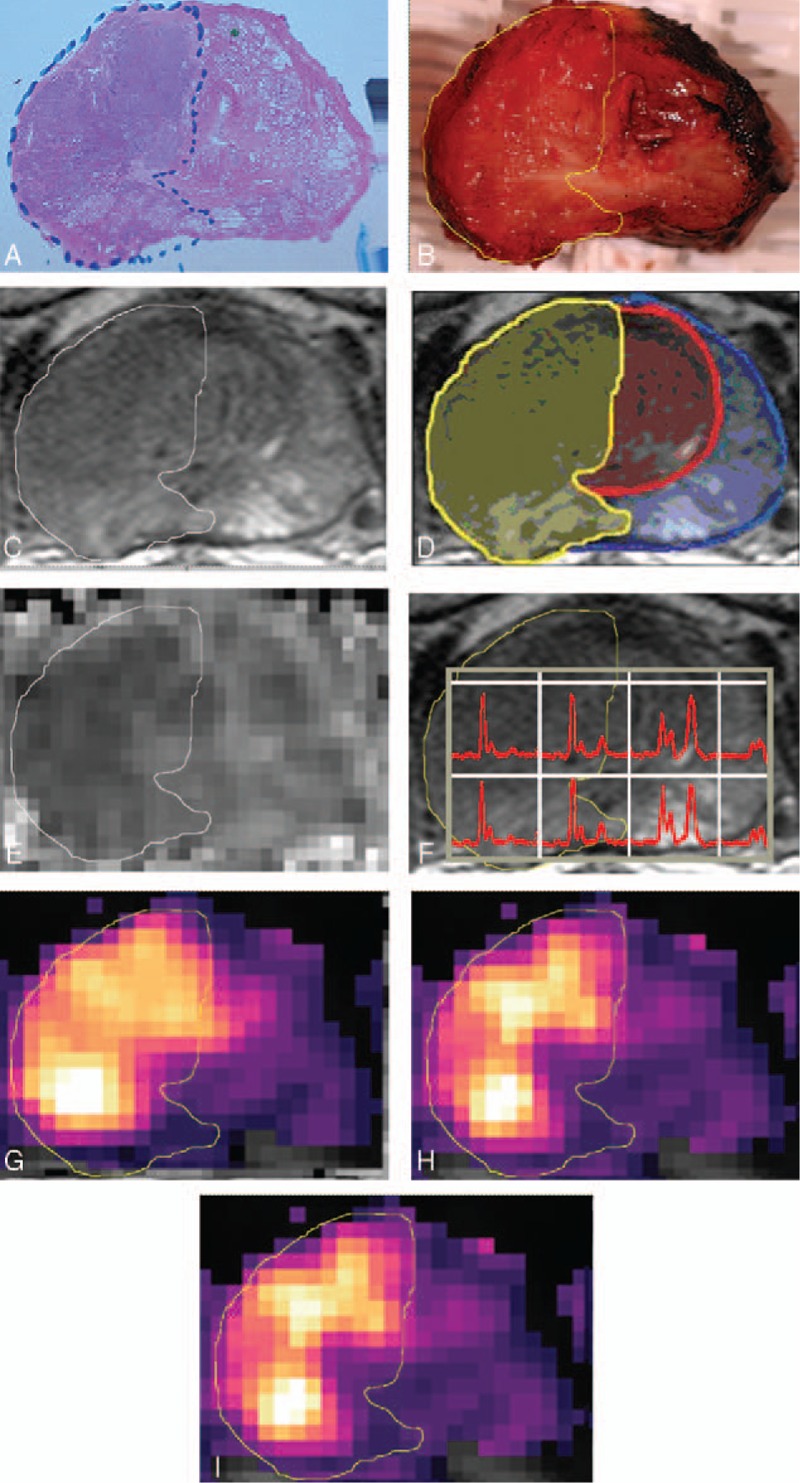
A 63-year-old man with tumor (Gleason grade, 4 + 3); prostate-specific antigen, 13.1 ng/mL in the right lobe of peripheral zone and central gland with no lymph node malignancy. A, Photomicrograph of prostatectomy specimen shows tumor outline. (H and E); B, Photomicrograph shows fresh slice of macroscopic specimen corresponding to A with tumor outline warped onto it. C, T2-weighted MR image shows tumor outline warped further to fit MRI slice. D, T2-weighted MR image shows outlines of whole prostate (blue) and central gland (red) with histologically defined tumor region (yellow) superimposed. E–I, Apparent diffusion coefficient map (E), MR spectroscopic voxels (F), and vascular parametric maps of initial area under the gadolinium plasma concentration-time curve (G), forward rate constant K^trans^ (H), and return rate constant k_ep_ (I). Reprinted with permission from the American Journal of Roentgenology.^58^

In quantifying the apparent diffusion coefficient (ADC, Figs. 2 and 3), nonlinearity of diffusion-encoding gradients may lead to bias in ADC estimates at the edges of large fields-of-view^11,17^ making sequential acquisition of multiple stations, with each station acquired at the isocenter, essential for larger volumes. The alignment of stations for whole-body DW-MRI may be improved by omitting shimming on each station and using the same center frequency at all stations.^12^ The optimal choice of b-values depends on SNR and the ADC of the tumor/tissue of interest.^18^ A minimum of 2 b-values is required for ADC estimation, but a larger number of appropriately chosen b-values is required for investigation of other models, which increases the acquisition time.^19^

Standard DW-MRI sequences average signal over respiratory cycles, with 3 to 6 signal averages being common. Breath-holding and respiratory triggering may produce sharper images, but respiratory triggering, using bellows or a navigator, has failed to show advantages over free-breathing in estimation of ADCs in abdominal organs.^20,21^ Cardiac triggering is not often used but may have value in DW-MRI in the upper abdomen.^22^ Peristaltic motion can be reduced by administration of an anti-peristaltic agent before imaging.

DCE-MRI is usually acquired using a 3D gradient echo sequence, with T_1_-weighted images (Fig. 2B). Proton density-weighted images acquired before, or in some cases after, contrast agent administration are employed in addition if quantitative analysis is required because pharmacokinetic modeling requires robust estimation of T_1_ relaxation times. A reproducible contrast-agent injection rate (usually 2 to 4 mL/s)^23^ is used and postcontrast images are acquired every 5 to 10 seconds in order to provide sufficient data support to model the gadolinium concentration-time curve. Postcontrast imaging for at least 5 minutes after injection is recommended.^23,24^

Quantitative analysis requires estimation of the arterial input function (AIF). Patient-specific AIFs can be estimated from the main DCE-MRI acquisition or from a pre-bolus acquisition. The former requires the presence of a suitable artery within the imaging volume, while the latter uses administration of a pre-bolus followed by dynamic imaging before and separate from the main DCE-MRI acquisition. Both techniques may be adversely influenced by partial volume effects, signal nonlinearity, B_1_ inhomogeneity, and inadequate temporal resolution.^25,26^

In anatomical regions that are affected by respiratory motion, for example, abdominal tumors, DCE-MRI data may be acquired using sequential breath-holds with breathing intervals between acquisitions.

MRS measurements provide information regarding the concentration (and sometimes other properties such as diffusion) of relatively abundant low molecular weight metabolites in a target volume (or volumes) of tissue (Figs. 1 and 3). MRS data are usually acquired from single voxels, single slices, or 3D volumes. For mpMRI, full 3D acquisition is usually most appropriate. For coupled spin systems, it is essential to select the acquisition echo time to achieve the optimal in-phase signals (eg, for lactate^27^ and citrate in prostate^28^). The repetition time should be chosen with reference to expected metabolite T_1_ to achieve optimal SNR per unit time.^29^

Similar principles apply to acquiring signals from other nuclei (^31^P, ^13^C, etc.), but require a coil combination that also permits acquisition at the ^1^H frequency. Although these coils often incorporate surface coil transmitters, relatively uniform spin excitation can still be achieved with sequences including adiabatic RF pulses to create uniform spin excitation.^30,31^

Historically, MR spectroscopic imaging studies were constrained by the time required to acquire data from all the phase-encoding steps. When the intrinsic SNR is high, faster acquisitions using parallel acquisition and echo-planar methods are possible so that whole brain coverage in approximately 20 minutes with 5 to 10 mm voxels is achieved.^32^

Most MRS measurements require care to eliminate unwanted signals. Several strategies exist for suppressing unwanted water and lipid signals^33–37^ or to excite metabolites of interest without exciting these unwanted signals.^38^ Some residual water signal, however, can be useful for subsequent frequency and phase correction of the data.^39,40^ Unwanted signals from outside the required volume can be reduced using very selective saturation slices^41^ or high-bandwidth excitation pulses in a sequence such as LASER.^42–45^ The latter methods also reduce the effects of the chemical shift displacement artifact (voxels of different metabolites being shifted relative to each other, and attenuation of signals from coupled spin systems^46^).

MRS data may often be compromised by tissue motion. A range of methods exist, including simple breath-holding, respiratory and cardiac triggering, and dynamic updating of scan parameters to minimize, follow, and correct for motion.^40,47–52^

## TECHNICAL CHALLENGES IN DATA ANALYSIS

Analysis of DCE-MRI, DW-MRI, and MRS data may be qualitative or quantitative, or in some cases “semi-quantitative.” Data analysis may be further categorized into online and offline methods. The former uses tools provided by the scanner manufacturers, either on the scanner or on a separate workstation or PACS system, for example, visual assessment of ADC maps, contrast agent uptake curves, and spectra. Offline methods use processing steps outside the clinical workflow and involve transfer of the data to a separate system.

Qualitative or semi-quantitative analyses, for example, those recommended by the Breast/Prostate Imaging and Reporting Systems (BI-RADS/PI-RADS),^53,54^ may be carried out online. They are quick, have a simplified workflow, and use validated systems and processes. Some quantitative analyses are also possible online [eg, estimation of ADC summary statistics from a region of interest (ROI)]. Offline analysis using in-house or third-party software (eg, quantitative analysis using pharmacokinetic modeling of DCE-MRI data^55^) may be time-consuming and may not be easily incorporated into the clinical workflow.

Most reported studies of quantitative mpMRI analyze each imaging method separately (Fig. 2), for example, estimating the median ADC from DW-MRI (Fig. 2A) and median K^trans^ from DCE-MRI (Fig. 2B), in some cases copying the same ROIs between DW-MRI and DCE-MRI, with assessment of the summary statistics separately or in combination.^56,57^ Thus, multi-parametric analysis on a per-pixel basis requires registration of images from each imaging sequence, including deformations if distortion has occurred and resampling if resolutions differ (Fig. 3). This is a challenging task, which is not routinely undertaken.^58^ More commonly, simple images showing parametric maps overlaid on anatomical images are used to aid data interpretation in a clinical setting (Figs. 2A, B).

### DW-MRI

On qualitative analysis, most solid tumors exhibit restricted diffusion with retention of signal intensity with increasing b-value compared with neighboring tissues, therefore appearing hyperintense on high b-value images and hypointense on ADC maps (Fig. 3E).^59,60^ To distinguish structures with long T_2_ relaxation times, for example, cystic or necrotic regions from solid tumor, it is essential to examine the ADC map in conjunction with high b-value images and T_2_-w images. Regions with long T_2_ may appear bright on high b-value images, so-called “T_2_ shine-through” effect, but can be distinguished by the absence of restricted diffusion on ADC maps. Maximum intensity projections (MIPs) of high b-value images using an inverted grey scale can be produced on the scanner console and are used particularly in whole-body DW-MRI for evaluation of diffuse^61^ and focal skeletal disease.^62,63^ Segmentation of the whole tumor burden has been shown to have value in evaluation of bone disease.^61,64,65^

The simplest level of quantitative analysis can be carried out using ROIs drawn using manufacturers’ tools or PACS systems. Offline analysis allows derivation of ADC summary statistics from volumes of interest (VOIs). A minimum lesion size of 1 to 2 cm should be imposed in selecting lesions for analysis to avoid partial volume effects. Good SNR is required to avoid noise bias in ADC estimates. Fitting alternative models to DW-MRI data, for example, bi-exponential, stretched exponential, or kurtosis models, requires offline processing and may provide additional information in some cases. Application of alternative models requires appropriately chosen b-values; it is also essential to avoid overfitting the data and consider the effects of noise. Although estimates of ADC and some other fitted parameters have shown good repeatability, estimates of *f* and D^∗^ from the bi-exponential model generally exhibit poorer repeatability.^66,67^

### DCE-MRI

Visual assessment of pre- and postcontrast images, or of curve shapes, can be carried out on the scanner console or separate workstation. Semi-quantitative analysis, for example, estimation of properties of the relative enhancement curves such as time-to-peak enhancement, maximum slope and peak enhancement, or characterization of curve shapes (persistent increase, plateau, or washout) may also be carried out on the same systems. These assessments are relatively simple to conduct and are clinically relevant, leading to their inclusion in BI-RADS and PI-RADS criteria.^53,54^ It is important to note, however, that semi-quantitative methods may be influenced by properties of the scanner or the injection procedure and may thus be difficult to make comparisons between patients or scanners.^23,24^

Quantitative analysis using pharmacokinetic modeling may provide valuable information related to perfusion and permeability, but the complexity of the offline analysis required and lack of consensus on methods and software has so far limited applications mainly to clinical trials in expert centers. A number of models are available^68^ and consensus recommendations list the transfer constant (K^trans^, Figs. 2B, 3H) and initial area under the gadolinium concentration time curve (IAUGC, Fig. 3G) as recommended primary end-points.^24^ Reliable estimation of the AIF from individual patient-based measurements is problematic and may contribute to the observed poor repeatability of fitted parameters. A population-based AIF improves repeatability and removes the requirement for an estimate of the AIF as part of the DCE-MRI acquisition.^25,26^

### MRS

Magnetic resonance spectra can be evaluated in a variety of ways. Sometimes visual inspection is sufficient. Some clinical questions can be adequately addressed by analyzing quantitative data from a single voxel representing the corresponding tissue. However, for probing lesion heterogeneity, grids of MRSI spectra need to be analyzed (Figs. 1D, 3F). Using high-resolution spectral grids [eg, a matrix of 100 (read) x 50 (phase) x 18 (slice) spatial samples for a field-of-view of 280 mm × 280 mm × 180 mm]^69^ with peak fitting and smoothing, images of individual metabolites can be obtained.

Quantitative methods of MRS analysis include measuring peak area ratios, such as (choline + creatine + polyamines)/citrate in ^1^H MR spectra of the prostate,^70^ or total phosphomonoester (PME)/ATP in ^31^P MR spectra of tumors.^71^ Lesions are usually characterized by metabolite ratios deviating from normal. However, such ratios depend upon many factors, in particular sequence timing, which makes it hard to compare data between institutions. By applying suitable correction factors, it is possible to produce estimates of metabolite concentration,^72–74^ although this usually relies on assumptions that are hard to verify (such as correct values for the relaxation time constants T_1_ and T_2_). Ideally, experimental design should aim to minimize dependence on these factors.

Obtaining peak areas from spectral data involves a number of steps, including phase correction (sometimes individually for each transient to overcome motion effects).^39^ Baseline correction has to make assumptions about macromolecules present.^75^ Spectral analysis tools on clinical scanners are limited and specialist spectral processing and analysis software, such as jMRUI^76,77^ and LCmodel,^78,79^ or other in-house software are often employed. More sophisticated tools provide estimates of uncertainty in the fit (usually Cramer-Rao Lower Bounds, although these need to be treated with some caution^80^ because they assume that the model is a good representation of the data, which is not always the case, and some method for quality control is required^81^). The results of spectral analysis generally cannot be imported to PACS systems and require separate viewing.

## CHALLENGES IN DATA INTERPRETATION

### Qualitative Image Evaluation

One of the greatest challenges of qualitative image interpretation arises from a need to combine a range of qualitative features from multiple image types and interpret them consistently. Driven by this need for consistency, structured qualitative (or semi-quantitative) scoring systems have been proposed. The most widely adopted of these are the BI-RADS and PI-RADS systems that use qualitative and semi-quantitative assessment of mpMRI to characterize lesions.^53,54^ These scoring systems express the likelihood of cancer based on mpMRI features where no parameter independently has sufficiently significant positive or negative predictive value for malignancy. On the basis of scores, patients are stratified for appropriate management; those at a higher risk of malignancy are directed toward tissue sampling.^82,83^

These semi-quantitative reporting systems express mpMRI in a consistent language and reduce variability between readers. Inter-observer variability is lower and clarity of communication among physicians improved for BI-RADS compared with unstructured reporting, with similar results seen for similar systems in other subspecialties.^84–86^ Structured qualitative mpMRI reporting also increases efficiency of data mining and correlation with histopathology, enabling performance feedback for radiologists and systematic reader training. The importance of training is demonstrated by stronger reporting agreement between experienced readers than between less experienced readers, for whom rates of identifying and sampling malignant lesions (ie, accuracy) improve as their training progresses.^87,88^ Despite the advantages of reducing errors with structured reporting, many radiologists are reluctant to adopt this practice outside specific clinical settings. There is a perceived work-flow disruption associated with “pro-forma” reporting as well as the limit of the available lexicon. Large randomized trials demonstrating outcome benefit for structured reporting are lacking and this area requires further investigation.^84,89^

### Repeatability and Reproducibility for Quantitative Studies

For quantitative biomarker evaluation, the Radiological Society of North America (RSNA) Quantitative Imaging Biomarkers Alliance (QIBA) has recommended that the uncertainty in a measurement must be evaluated before use in therapy response evaluation, prognostication, or lesion characterization.^90^ At a minimum, this includes analysis of marker precision and bias estimation, along with measurement linearity^91^ by comparison with an accepted reference or standard measurement. For many MRI measurements, in vivo physiological reference is not available and bias/linearity measurements are extrapolated from phantom studies.^90–92^ It is worth noting that qualitative scoring systems (eg, BI-RADS/PI-RADS) yield categorical values, for which absolute differences or ratios between 2 measurements are not meaningful and reference standards do not apply.^90^

Repeatability is defined as the closeness of agreement between measured values obtained by replicate measurements performed on the same subject, on the same scanner, with identical imaging protocols. In clinical MRI studies, repeatability is usually estimated through test-retest measurements, for example, 2 MRI examinations carried out with a short interval of separation.^90^ Reproducibility, on the other hand, is the closeness of agreement between measured values obtained by replicate measurements under different conditions, which may include different scanners or operators.^90^ Differences between scanners (or institutions), imaging readers, imaging protocols, or postprocessing methods contribute to imperfect reproducibility of quantitative results.^91^ It is usually possible to perform imaging for each patient on the same scanner and for analysis to be carried out by the same reader, especially within single-center studies and clinical trials. Factors affecting reproducibility will, however, include intra- (and inter-) observer variability, arising from factors such as lesion segmentation,^90,93^ and may also include differences between scanners or imaging protocols^11,94,95^ and analysis software^96^ in multicenter studies.

It is unlikely that quantitative analysis will obviate the need for qualitative image interpretation, especially when delineating tumor from surrounding anatomical structures for the purpose of surgical planning. It is, however, likely that improved probing of biophysical processes using quantitative images of tumors will become increasingly valuable in clinical trials and for directing therapy in clinic, especially with increased availability of targeted therapeutic agents.^97^

## VALUE OF mpMRI IN CLINICAL DECISION-MAKING

To be of use in clinical decision-making, biomarker(s) must improve disease detection, aid staging, or provide prognostic information or robust response assessment and follow-up. Disease detection and staging are usually done by qualitative, subjective assessment of images, whereas prognostic or response assessment biomarkers require quantitative evaluation. The evidence for the use of DW-MRI alone in cancer diagnosis is overwhelming with nearly 1000 publications and numerous meta-analyses in the last decade advocating its use in a variety of tumor types.^98–101^ Diagnostic accuracies vary by disease site, but in most meta-analyses, sensitivity and specificity were greater than 80%, except for prostate cancer, wherein pooled sensitivity for DW-MRI in a meta-analysis of 21 studies was 62%^102^ and in breast cancer wherein specificity at best is around 71%.^103^ In both these tumor types, therefore, there has been a move to use a combination of parameters to improve diagnostic performance and an increasing body of data are accruing indicating the superiority of a multi- over a single parameter approach (Fig. 3). In the brain, wherein magnetic field homogeneity is good and SNR high, quantitation is more robust, so mpMRI has been exploited more fully as a prognostic biomarker as well as for assessment of treatment response (Fig. 1).

### Prostate Cancer

In detecting prostatic cancer, mpMRI evaluated using a PI-RADS system has proven of benefit in peripheral zone (PZ) but not transitional zone (TZ) lesions.^104^ Where no distinction is made between PZ and TZ, DW-MRI has the highest sensitivity for tumor localization (31.1% for T_2_-w vs 27.4% for DCE-MRI and 44.5% for DW-MRI) but combining all 3 techniques improved sensitivity to 58.8%.^105^ The performance of mpMRI also depends on the grade of cancer. mpMRI outperforms clinical risk calculators for predicting high-grade prostate cancer (AUC 0.769 vs 0.676, respectively)^106^ and has proven to be of benefit when added to clinical criteria for detecting these lesions.^107^ Not surprisingly, therefore, the diagnostic performance of mpMRI in a cohort of 100 patients proved better for Gleason grade >7 than <7 tumors and tumors >1 cm^3^ than those 0.5 to 1.0 cm^3^.^108^ Recognition of an abnormality on mpMRI allows targeted biopsy of the suspicious area either through cognitive fusion of the MR images with the transrectal ultrasound (TRUS) images or by registration and overlay of the MR data on the real-time ultrasound, which requires specialist software. There is now an increasing body of evidence indicating that targeted biopsies increase the sensitivity of prostate cancer diagnosis^109^ compared with systematic biopsies alone. Interestingly, disease identification on contrast-enhanced imaging is associated with increasing lesion size, intermixed benign epithelium, loose stroma, and high malignant epithelium to stroma ratio, while on DW-MRI only size, Gleason score and loose stroma were significant for lesion identification.^110^

For disease staging, semi-quantitative assessment using PI-RADS scoring is used. Its sensitivity for detecting extracapsular extension (ECE, Stage 3a) in patients undergoing prostatectomy is only 35% to 49%,^111,112^ although specificity is high (90% and 74%, respectively). For seminal vesicle invasion, a 65% sensitivity (Stage 3b, verified on pathology at biopsy and subsequently at prostatectomy) is recorded.^113^ The addition of mpMRI evaluation to clinical nomograms (Partin tables) improves sensitivity of detecting ECE on pathology to 84% (positive predictive value [PPV] 66.7%, negative predictive value [NPV] 94.9%).^114^ PPV is better in the clinically defined (D’Amico criteria) high-risk groups at 88.8%, while NPV is best in those at low risk (87.7%).^115^ A regression model for predicting ECE showed that the most reliable predictors are DW-MRI + DCE-MRI and Gleason score.^116^

Qualitatively scored mpMRI has also been used to predict biochemical recurrence in a population of >300 cases undergoing radical prostatectomy,^117^ but the clinical utility of this relies on the ability to change adjuvant therapy protocols.

### Breast Cancer

In breast cancer, the addition of normalized ADCs to 3D T_1_-w and DCE-MRI data improves diagnostic performance (AUC 0.98 vs 0.89).^118^ The value of parameter combinations has been confirmed in other studies: analysis of 100 breast lesions (27 malignant and 73 benign) in 77 women showed that ADC is lower for lesions exhibiting predominantly washout or plateau patterns than those exhibiting predominantly persistent enhancement, and in multivariate analysis, worst curve type and ADC were significant independent predictors of malignancy.^119^ Extension of mpMRI to 3 parameters (DCE-MRI, DW-MRI, and 3D ^1^H-MRSI) rather than 2 (DCE-MRI and DW-MRI) showed that the former yielded significantly higher areas under the curve than histology (0.936 vs 0.808) because of elimination of false-negative lesions and reduction in false-positives.^120^

Seven features derived from DW-MRI and DCE-MRI (eg, slope, entropy, ADC) have been shown to discriminate malignant from benign lesions and their combination achieves the highest classification accuracy.^121^ The use of multiple parameters from DCE-MRI alone has illustrated the possibilities of identifying intrinsic imaging phenotypes of breast cancer based on hierarchical clustering of extracted feature vectors. These features have been linked to risk of recurrence based on gene expression.^122^

As with prostate cancer, mpMRI has been explored for disease staging albeit more modestly. A meta-analysis of 624 breast cancer patients from 9 eligible cohort studies, 254 of whom had lymph node metastases (LNMs) and 370 who did not, suggested that ADC values in patients without LNM were higher.^123^ A more pressing need in breast cancer is in prognostication, and efforts here have exploited higher field strengths (7 T) to obtain quantitative mpMRI data. A sensitivity of 100%, specificity of 88.2%, has been claimed for a combination of DW-MRI + DCE-MRI, which was greater than for individual parameters (DCE-MRI 100%/53.2%; DW-MRI 93.1%/88.2%) such that it eliminated all false-negative findings and reduced false-positive findings.^124^ The addition of ^31^P-MRS in a small (n = 15) study by another group showed an inverse relationship between ADC and tumor grade. A relative increase of PME over phosphodiester (PDE) showed significant association with increasing mitotic counts.^125^

An important niche for quantitative mpMRI in breast cancer is in response assessment to neoadjuvant chemotherapy (NAC). DCE-MRI + DW-MRI has been shown to have a higher specificity (80.0%), accuracy (91.0%), and PPV (93.2%) than DCE-MRI or DW-MRI alone.^126^ This was confirmed in a response assessment study wherein DCE-MRI and DW-MRI data acquired before (n = 42) and after 1 cycle (n = 36) of NAC showed that the k_ep_/ADC after the first cycle of NAC discriminated patients who went on to achieve a pathological complete response and achieved a sensitivity, specificity, PPV, and area under the receiver operator curve (AUC) of 0.92, 0.78, 0.69, and 0.88 respectively, which were superior to the single parameters k_ep_ (AUC, 0.76) and ADC (AUC, 0.82).^57^ MRS has also been exploited in this regard and showed larger reductions in choline SNR (35% vs 7%) in pathological complete or partial responders compared with nonresponders after 1 cycle of chemotherapy.^127^

### Gliomas

The use of morphological MRI with DCE-MRI and DW-MRI for gliomas has been part of clinical practice for more than a decade. Refinements include quantitation to improve tumor grading. Relative cerebral blood volume (rCBV) alone in a study of 56 patients gave a sensitivity and specificity of 100% and 88% and addition of DW-MRI and MRS improved the specificity to 96%.^69^ Using a radiological progression index derived from MR spectroscopy and MR perfusion showed that the cumulative data were able to classify the patients into different grades and were predictive of overall survival: MR hyperperfusion indicated a shorter survival for diffuse intrinsic pontine glioma patients.^128^ Further advances exploiting statistical features obtained from the parametric maps in a prospective study of 74 glioma patients showed that the presence/absence of enhancement coupled with the kurtosis of the first-pass curve was the feature combination that best predicted tumor grade with the presence/absence of enhancement being the more important feature.^129^

Quantitation has also proved promising in a prognostic context: in a single-center study of 56 patients, the simultaneous analysis of ADC and rCBV 3 weeks after completion of radiation and concurrent temozolomide improved the predictive potential for patient survival compared with the single parameters.^130^ These kind of data are also proving worthwhile in a pediatric population wherein increased choline to NAA and increased perfusion on dynamic susceptibility contrast MRI (DSC-MRI) at baseline each predicted shorter survival in children with diffuse pontine glioma, and increased perfusion measured at any time-point in treatment also predicted shorter survival.^131^

### Other Cancers

In cancers other than those above, the use of mpMRI has mainly been in the assessment of response to therapy. In high-grade soft-tissue sarcomas, qualitative assessment of both DCE-MRI and DW-MRI performed better than standard morphological imaging for predicting response to NAC as verified at subsequent surgery, although the functional images were not assessed in combination.^132^ A meta-analysis of 43 rectal cancer studies, 30 of which included DW-MRI and 13 DCE-MRI, indicated that both these techniques showed additional value in the prediction and detection of complete response to therapy compared with conventional T_2_-w sequences alone.^133^ An ongoing multicenter trial in Australia is focusing on the prospective evaluation of quantitative mpMRI (ADC, K^trans^, k_ep_) to stratify patients and guide radiation dosing.^134^ The combination of T_2_-w + DW-MRI + DCE-MRI has been shown to be of benefit for distinguishing low- from high-grade bladder cancers (100% sensitivity, 95% specificity in 49 T1 and T2 lesions),^135^ while the combination of wash-in and wash-out ratios on DCE-MRI together with ADC has been shown to be useful in renal clear cell cancers wherein higher Fuhrman tumor grades had lower parenchymal wash-in indices and lower ADCs than low-grade lesions.^136^ There has been less demand for mpMRI for detecting and grading other cancers, as single parameters often suffice or treatment paradigms do not warrant the inclusion of complex methodology in the patient's diagnostic pathway.

## FUTURE DIRECTIONS

To date, the major applications of multi-parametric imaging in the clinic have been through a qualitative approach using radiologist scores of standardized consensus systems such as PI-RADS and BI-RADS. An obvious advance would be to increase use of quantitative techniques to their full potential. However, methods of quantitation are variable, lengthy, and may introduce error. These are overriding disadvantages when planning patient management. An equivalence between qualitative scoring and a fully quantitative approach in prostate cancer assessment has been demonstrated,^58^ so that very large economic benefits in quantitative assessments would be needed before clinical adoption. An area wherein they may be beneficial is in understanding tumor biology, where discordance between techniques may provide an understanding with underlying histopathology, which could then be clinically translated. Another area of development is in fusion of multimodality data sets. With ultrasound elastography providing measures of tissue stiffness^137^ and PET studies mapping metabolism, hypoxia, and tumor-specific antigens and receptors,^138^ it will be possible to adopt a multi-parametric, multimodality approach to more accurately characterize and monitor tumor behavior in the clinic to deliver individualized treatment plans.
